# Breakthrough Mucormycosis Developing on Mucorales-Active Antifungals Portrays a Poor Prognosis in Patients with Hematologic Cancer

**DOI:** 10.3390/jof7030217

**Published:** 2021-03-17

**Authors:** Dierdre B. Axell-House, Sebastian Wurster, Ying Jiang, Andreas Kyvernitakis, Russell E. Lewis, Jeffrey J. Tarrand, Issam I. Raad, Dimitrios P. Kontoyiannis

**Affiliations:** 1 Department of Infectious Diseases, Infection Control, and Employee Health, The University of Texas MD Anderson Cancer Center, Houston, TX 77030, USA; dbaxell@mdanderson.org (D.B.A.-H.); stwurster@mdanderson.org (S.W.); yijiang@mdanderson.org (Y.J.); Andreas.Kyvernitakis@ahn.org (A.K.); russeledward.lewis@unibo.it (R.E.L.); iraad@mdanderson.org (I.I.R.); 2 Section of Infectious Diseases, Department of Medicine, Baylor College of Medicine, Houston, TX 77030, USA; 3 Department of Laboratory Medicine, The University of Texas MD Anderson Cancer Center, Houston, TX 77030, USA; jtarrand@mdanderson.org

**Keywords:** mucormycosis, mortality, antifungal therapy, breakthrough mold infection, hematologic malignancy

## Abstract

Although breakthrough mucormycosis (BT-MCR) is known to develop on mold-active antifungals without Mucorales activity, it can also occur while on Mucorales-active antifungals. Herein, we retrospectively compared the characteristics and outcomes of patients with hematologic malignancies (HMs) or hematopoietic stem cell transplant (HSCT) who developed BT-MCR on mold-active antifungals with or without Mucorales activity. Of the patients developing BT-MCR, 16 were on Mucorales-active antifungals (9 isavuconazole, 6 posaconazole, 1 amphotericin B), and 87 were on other mold-active agents (52 voriconazole, 22 echinocandins, 8 itraconazole, 5 echinocandin + voriconazole). Both groups were largely comparable in clinical characteristics. Patients developing BT-MCR while on Mucorales-active antifungals had higher 42-day mortality, from either symptom onset (63% versus 25%, *p* = 0.006) or treatment initiation (69% versus 39%, *p* = 0.028). In multivariate Cox regression analysis, exposure to Mucorales-active antifungals prior to BT-MCR had a hazard ratio of 2.40 (*p* = 0.015) for 42-day mortality from treatment initiation and 4.63 (*p* < 0.001) for 42-day mortality from symptom onset. Intensive care unit (ICU) admission and APACHE II score at diagnosis, non-recovered severe neutropenia, active HM, and amphotericin B/caspofungin combination treatment were additional independent predictors of 42-day mortality. In summary, BT-MCR on Mucorales-active antifungals portrays poor prognosis in HM/HSCT patients. Moreover, improvements in early diagnosis and treatment are urgently needed in these patients.

## 1. Introduction

Invasive mucormycosis (MCR), caused by molds of the order Mucorales, has emerged as the second most common invasive mold infection in patients with hematologic malignancies (HMs) and recipients of hematopoietic stem cell transplants (HSCTs) [1]. Mortality from invasive MCR in patients with HM and recipients of HSCT can be as high as 80% [1,2]. In view of their severe immunosuppression and risk for opportunistic invasive fungal infections, these patients routinely receive primary or secondary antifungal prophylaxis, mostly with mold-active triazoles [3,4].

Breakthrough mucormycosis (BT-MCR), or MCR that is diagnosed while a patient is receiving antifungal medications, classically occurs in patients receiving mold-active but non-Mucorales-active antifungal therapy, most commonly voriconazole [2] or echinocandins [5]. However, BT-MCR has also been known to sporadically occur in patients receiving Mucorales-active antifungals such as posaconazole [6], isavuconazole [7], or even amphotericin B [8]. Thus far, the characteristics and prognosis of patients diagnosed with BT-MCR developing on Mucorales-active antifungals have been scarcely described. Therefore, we sought to evaluate and compare the presentation and prognosis of patients with BT-MCR on mold-active antifungals with and without Mucorales activity.

## 2. Patients and Methods

### 2.1. Study Design

We conducted a retrospective chart review of all patients with HMs and/or recipients of HSCTs who were diagnosed with invasive MCR at the University of Texas MD Anderson Cancer Center (MDACC) between April 2000 and April 2020. We included patients who were ≥18 years old, met the European Organization for Research and Treatment of Cancer/National Institute of Allergy and Infectious Diseases Mycoses Study Group (EORTC/MSG) criteria for proven or probable MCR [9], and had breakthrough infection according to the definitions proposed by the MSG/European Confederation of Medical Mycology (ECMM) [10]. Patients were categorized into one of two groups based on the mold-active antifungal medication they were receiving prior to diagnosis of BT-MCR: Mucorales-active antifungals consisting of isavuconazole, posaconazole, or (lipid formulation) amphotericin B, or other mold-active antifungals (with no Mucorales activity), consisting of echinocandins, itraconazole, or voriconazole. Our analysis did not include any patients with BT-MCR on the mold-inactive triazole fluconazole or the very uncommon patients with de novo MCR (no prior antifungal exposure). Antifungals were selected for prophylaxis at the discretion of the treating oncologist. The study was approved by the MDACC institutional review board.

### 2.2. Characteristics Assessed and Definitions

We extracted the following patient demographics and clinical characteristics: age, gender, race, Hispanic/Latinx ethnicity, underlying malignancy (categorized as leukemia/myelodysplastic syndrome or lymphoma/myeloma), malignancy status (active or in remission), HSCT status, presence and status of graft-versus-host disease (GvHD), presence and duration of neutropenia, neutrophil recovery after neutropenia, corticosteroid use, history of diabetes mellitus, site/type of MCR, etiological organism of MCR, Acute Physiology And Chronic Health Evaluation II (APACHE II) score at diagnosis, intensive care unit (ICU) admission at diagnosis or anytime during treatment, initial antifungal treatment strategy, time from symptom onset to change of antifungal treatment, and surgical management.

Neutropenia was defined as absolute neutrophil count (ANC) ≤ 500 cells/μL. Neutrophil recovery was defined as the occurrence of 3 consecutive days of ANC > 500 cells/μL after the diagnosis of neutropenia. Corticosteroid use was defined as prednisone 600 mg or dose-equivalent received in the 30 days preceding the diagnosis of BT-MCR. Type of infection was identified as localized (single-location soft tissue involvement or isolated organ system not involving the sinopulmonary tract), sinopulmonary, or disseminated (involvement of two or more non-contiguous sites). The initial treatment strategy was defined as any Mucorales-active regimen administered in the first seven days of treatment [11] that was not identical to the pre-infection regimen.

### 2.3. Outcomes and Statistical Analysis

The primary outcomes were 42-day all-cause mortality (a) from time of Mucorales-active treatment initiation and (b) from onset of symptoms. Additional secondary outcomes included 84-day all-cause mortality and median time to death from treatment initiation or onset of symptoms.

Categorical variables were compared by chi-square or Fisher’s exact test. Continuous variables were compared by Wilcoxon rank sum test. The Cox proportional hazards regression model was used to identify factors that were independently associated with mortality outcomes, which were reported with hazard ratios and 95% confidence intervals. The following factors were included in the analysis: patients’ demographic and clinical factors at baseline, medical history, neutropenia and recovery from neutropenia, presence of GvHD, antifungal prophylaxis/therapy prior to BT-MCR diagnosis, disease severity including ICU status and APACHE II score at diagnosis, as well as infection and treatment variables including surgical treatment and antifungal therapy. First, univariate analysis was performed for each individual factor. Next, all variables with *p*-values ≤ 0.25 on their univariate analyses were included in a full Cox regression model. The full model was then reduced to the final model by a backward elimination procedure so that all factors remaining in the final model had *p*-values ≤ 0.05. The proportional hazards assumption of the Cox regression model was assessed using a time-dependent covariate method. When evaluating mortality since symptom onset, treatment was considered as a time-dependent variable.

Survival curves were estimated by the Kaplan–Meier method and survival probabilities were compared by the log rank (Mantel–Cox) test. All statistical tests were two-sided with a pre-set significance level of 0.05. Data analysis was performed using SAS version 9.3 (SAS Institute Inc., Cary, NC, USA). Kaplan–Meier curves were generated with GraphPad Prism 8 (GraphPad Software, Inc., San Diego, CA, USA).

## 3. Results

### 3.1. Patients and Clinical Characteristics

We identified 103 patients who were receiving mold-active antifungals as treatment or prophylaxis at the time that they were diagnosed with BT-MCR. Eighty-seven patients developed BT-MCR while on voriconazole (*n* = 52), echinocandins (*n* = 22), itraconazole (*n* = 8), or caspofungin/voriconazole combination (*n* = 5). Sixteen patients developed BT-MCR while on the Mucorales-active antifungals isavuconazole (*n* = 9), posaconazole (*n* = 6), or amphotericin B (*n* = 1).

Patient demographics and clinical characteristics of BT-MCR are provided in Table 1. The median age was 52 (range 18–76). Sixty-seven patients (65%) were male and 86 (84%) were white. The majority of patients had leukemia or myelodysplastic syndrome (MDS, 89%) and had active malignancy (78%). Fifty patients (49%) had been recipients of allogeneic HSCT, with 80% of them developing (GvHD). Sixty-five patients (63%) were neutropenic at diagnosis, with a median neutropenia duration of 21 days (interquartile range, IQR, 12–52) preceding BT-MCR. Forty-one (63%) of the patients who were neutropenic at the time of BT-MCR diagnosis had neutrophil recovery. Overall, thirty-six patients (35%) had received prior corticosteroids and 42 (41%) had a history of diabetes mellitus. The most frequent site of infection was sinopulmonary, and *Rhizopus* spp. was the most frequent genus isolated from patients (59%). The median APACHE II score at diagnosis as a measure of illness severity was 14 (IQR 12–17); 13% of the patients were in the ICU at the time of BT-MCR diagnosis, and 77% were admitted to the ICU or transitioned to hospice care at some point during BT-MCR treatment.

The most frequently employed initial treatment strategy was combination therapy, used in 83% of patients, with the most common combination being lipid-formulation amphotericin B, caspofungin, and posaconazole (30% of all patients). The median period from symptom onset to treatment initiation was 6 days (IQR 3–11); of note, this included patient-reported symptomatic days prior to the patient’s presentation to care.

When compared to patients receiving other mold-active antifungals, fewer patients with BT-MCR occuring while on Mucorales-active antifungals had a history of diabetes mellitus (13% vs. 46%, *p* = 0.012) or received surgical intervention as treatment for MCR (19% vs. 51%, *p* = 0.019). Otherwise, there were no significant differences between the two groups on univariate analysis (Table 1).

Additional clinical characteristics of the 16 patients who developed BT-MCR while on Mucorales-active antifungals (isavuconazole in 9, posaconazole in 6, amphotericin B in 1) are provided in Table 2. Most of these patients (10/16) were receiving Mucorales-active antifungals for primary prophylaxis. Five additional patients were receiving Mucorales-active antifungals for presumed mold pneumonia of unknown etiology and one patient for *Alternaria* infection. Three out of 6 episodes of BT-MCR on posaconazole occurred in patients who were taking posaconazole suspension (Table 2). Of these, only one had a posaconazole serum level drawn, which was low (<700 ng/dL). The posaconazole serum levels of the remaining three patients with BT-MCR on posaconazole tablets ranged from 1750 to 2520 ng/dL (Table 2). No serum levels were available for the patients with BT-MCR on isavuconazole. Antifungal in vitro susceptibility testing was not performed on the Mucorales isolates from these 16 patients. Of note, 12 out of 16 BT-MCR cases (75%) occurring while on Mucorales-active antifungals were identified in the last 5 years of the study (Table 2). For comparison, only 23 out of 87 BT-MCR cases on other mold-active antifungals occurred in the last five years (26%, data not shown).

### 3.2. Outcomes

**Figure 1:**
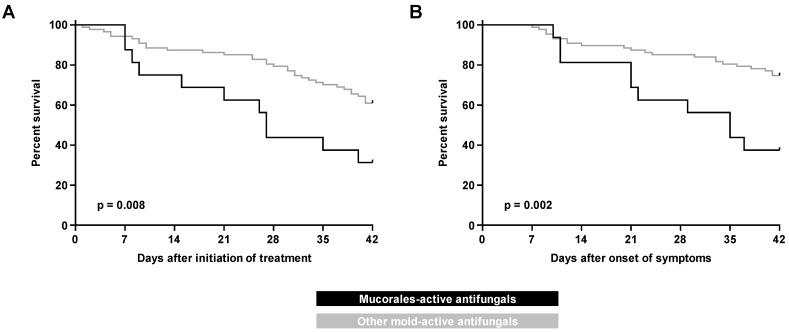
Kaplan–Meier curves of progression to 42-day mortality in patients with hematologic malignancy and recipients of hematopoietic stem cell transplants with breakthrough mucormycosis on Mucorales-active versus other mold-active antifungals as measured from (**A**) initiation of treatment and (**B**) onset of symptoms.

Overall, 32 patients (31%) died within 42 days of BT-MCR symptom onset, and 45 patients (44%) died within 42 days of starting treatment for BT-MCR (59% and 62%, respectively, for 84-day mortality). In the univariate analysis, 42-day mortality from both treatment initiation (69% vs. 39%, *p* = 0.028) and symptom onset (63% vs. 25%, *p* = 0.006) were higher in the Mucorales-active therapy group than in patients on other mold-active antifungals (Table 3). These results were corroborated by Kaplan–Meier survival analysis (Figure 1). The 84-day mortality outcomes from both treatment initiation and symptom onset were also worse in the Mucorales-active therapy group (Table S1, Figure S1). Similarly, median time to death from treatment initiation was shorter in the Mucorales-active therapy group (27 days, IQR 12–50) compared to that in patients with BT-MCR on other mold-active antifungals (49 days, IQR 30–146) (*p* = 0.007). This also held true for time to death from symptom onset (Table 3). There were no significant differences in mortality outcomes between individual antifungals within the two cohorts (Figure S2); however, the power of this analysis was limited by small sample sizes.

The Cox regression model was used to identify independent predictive factors for mortality in patients with a diagnosis of BT-MCR. Consistent with the univariate analysis, exposure to Mucorales-active prophylaxis or therapy prior to BT-MCR diagnosis had a hazard ratio (HR) of 2.40 (95% confidence interval, CI, 1.19–4.86, *p* = 0.015) for all-cause 42-day mortality from time of treatment initiation and a hazard ratio of 4.63 (95% CI 1.91–11.23, *p* < 0.001) from time of symptom onset (Table 4). APACHE II score and ICU admission at diagnosis were additional independent predictors of 42-day mortality from both time of treatment initiation (APACHE II: HR 1.21 per 1-unit increase, 95% CI 1.12–1.30, *p* < 0.0001; ICU: HR 2.46, 95% CI 1.07–5.68, *p* = 0.034) and symptom onset (APACHE II: HR 1.13 per 1-unit increase, 95% CI 1.04–1.23, *p* = 0.005; ICU: HR 4.71, 95% CI 2.05–10.85, *p* < 0.001) (Table 4). Another independent predictor of mortality within 42 days of symptom onset was treatment of BT-MCR with amphotericin B/caspofungin combination therapy (HR 8.15, 95% CI 3.09–21.48, *p* < 0.0001) (Table 4).

**Figure 2:**
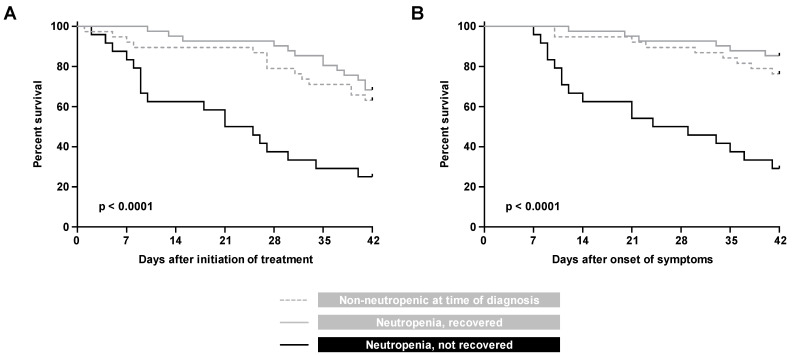
Kaplan Meier curves of progression to 42-day mortality from (**A**) initiation of treatment and (**B**) onset of symptoms, in patients with hematologic malignancy and recipients of hematopoietic stem cell transplants who were non-neutropenic at the time of BT-MCR diagnosis, neutropenic at diagnosis with subsequent neutrophil recovery, and neutropenic at diagnosis without neutrophil recovery.

Expectedly, lack of neutrophil recovery from neutropenia during treatment emerged as a strong predictor of 42-day mortality after BT-MCR treatment initiation (HR versus non-neutropenic patients 3.25, CI 1.53–6.90, *p* < 0.001) and symptom onset (HR 9.63. CI 3.57–25.99, *p* < 0.0001). This observation was corroborated by Kaplan–Meier analysis, confirming significantly heightened mortality of patients with non-recovered neutropenia, as compared to both non-neutropenic patients and patients who recovered from severe neutropenia during treatment of BT-MCR (*p* < 0.0001, Figure 2).

All predictors of 42-day mortality persisted on assessmemt of 84-day mortality from symptoms onset and treatment initiation (Table S1). Additionally, active cancer status was identified as an independent preditor of 84-day all-cause mortality as measured from both treatment initiation for BT-MCR (HR 2.50, 95% CI 1.01–6.21, *p* = 0.048) and symptom onset (HR 2.81, 95% CI 1.06–7.48, *p* = 0.039) (Table S1).

Notably, all-cause mortality at day 42 and day 84 in patients with BT-MCR did not improve over the observation period between 2000 and 2020. On the contrary, in the past five years, there has been a trend toward worse 84-day outcomes of BT-MCR from both initiation of treatment (*p* = 0.073) and symptom onset (*p* = 0.075) (Figure S3).

## 4. Discussion

We report the first study evaluating a sizeable cohort of patients with BT-MCR developing on Mucorales-active antifungal agents. Despite the frequent use of broad-spectrum antifungals with Mucorales activity, BT-MCR on Mucorales-active antifungals accounted for 34% of all BT-MCR cases at our institution in the last 5 years. This emphasizes the importance of continuous vigilance for MCR and avoidance of a false sense of security, even for patients on Mucorales-active antifungal prophylaxis and therapy.

Most patients with BT-MCR, including the ones with MCR developing on Mucorales-active antifungals, had significant burden of immunosuppression and active HMs. Corticosteroid use and diabetes mellitus, both known risk factors for MCR, were also common in our cohort. The acuity of illness in our patient population was reflected by the APACHE II score at BT-MCR diagnosis and by the fact that ICU or hospice admission during MCR treatment were common (Table 1). Extensive infection, typically sinopulmonary or disseminated disease, was seen in >80% of patients with BT-MCR. *Rhizopus* was the most common causative genus of BT-MCR in our cohort, consistent with prior reports [11,12,13].

The all-cause 42-day mortality of the entire cohort of patients with BT-MCR was consistent with our previously published experience [12]. Notably, the subgroup of 16 patients who received posaconazole, isavuconazole, or amphotericin B preceding BT-MCR diagnosis had distinctly worse outcomes for all-cause 42-day and 84-day mortality determined from both treatment initiation and symptom onset. BT-MCR on Mucorales-active antifungals was also found to be an independent predictor of mortality in Cox regression analysis (Table 4 and Table S1). This association has not previously been reported and invites further studies on the likely multifactorial, interrelated, and poorly understood etiological factors. For example, the hypothesis that patients developing BT-MCR on Mucorales-active antifungals had more profound immune deficits was not borne out by our data. In fact, there were no distinct differences in baseline demographic characteristics between patients developing BT-MCR on Mucorales-active antifungals compared to those with BT-MCR on other mold-active antifungals. In particular, both patient groups did not significantly differ in the occurrence or duration of neutropenia or frequency of allogenic HSCT recipients developing GvHD. Similarly, APACHE II scores, frequencies of disseminated MCR, and rates of ICU admission at the time of BT-MCR diagnosis were comparable in both cohorts, indicating that differences in 42- and 84-day all-cause mortality were not due to disparate infection severity at baseline. Furthermore, the significantly higher rate of surgical treatment in patients with BT-MCR who received agents without Mucorales activity (Table 1) was not found to be an independent predictor of all-cause mortality in multivariate Cox regression analysis. Collectively, these observations indicate that BT-MCR on Mucorales-active antifungals is a robust independent risk factor for mortality that was not confounded by differences in baseline characteristics or treatment variables between the two groups. Nonetheless, we cannot preclude that subtle differences in underlying risk factors such as iron overload or polymorphisms in immune signaling, which were not recorded in this study, might have contributed to disparate outcomes.

Of note, the distribution of the causative Mucorales genera was not different in the two cohorts, reflecting a lack of antifungal selection of more resistant Mucorales such as *Cunninghamella* spp. in patients with BT-MCR on Mucorales-active antifungals. Whether certain triazoles cause subtle changes to mucoralean virulence deserves further study. Interestingly, 10 of the 16 patients with BT-MCR on Mucorales-active antifungals were exposed to isavuconazole. Experimental data in flies indicate that isavuconazole may increase the virulence of Mucorales-order molds, although further validation in mammalian models is needed [14].

Additional independent risk factors for mortality in our analysis were severe neutropenia without neutrophil recovery during BT-MCR therapy, ICU admission at diagnosis, and active cancer status, as previously described [11,12]. Notably, combination therapy with amphotericin B and caspofungin appeared to portray worse prognosis, consistent with our prior experience [12], although this observation can be an artifact of small numbers, among other confounders. In a case–control study of MCR in solid organ recipients, Singh et al. reported inferior outcomes for combination therapy (amphotericin B and posaconazole) compared to that for amphotericin B monotherapy [15].

Our study had several limitations in view of its retrospective nature, the long review period, and the heterogeneous group of patients. We report all-cause mortality as the outcome, since assessing the attributable mortality is prohibitively difficult in the setting of competing causes of death in this very ill population at an institution with a low autopsy rate [16]. In addition, we did not provide in vitro susceptibility data in our cohort; nevertheless, the in vivo–in vitro correlation of outcomes in mold infections remains problematic [17]. Despite the overall size of our cohort, the group of patients with BT-MCR on Mucorales-active antifungals was small, which prevented performance of a propensity score analysis to better characterize factors affecting mortality compared to patients with BT-MCR on other mold-active antifungals. Finally, our results, although intriguing, cannot be generalized to other host groups at risk for BT-MCR (e.g., solid organ transplant recipients) who frequently receive mold-active prophylaxis.

## 5. Conclusions and Outlook

BT-MCR remains a significant threat to patients with HMs and recipients of HSCTs, and BT-MCR that develops on Mucorales-active antifungal therapy signifies a poor prognosis. As is the case for other opportunistic mold infections such as invasive aspergillosis [18] and fusariosis [19], we found that lack of neutrophil recovery is associated with poor prognosis of MCR in patients with hematologic cancer, irrespective of antifungal drug exposure. Further studies are needed to examine the use of investigational drugs with novel mechanisms of action against Mucorales [20] (as monotherapy or in combination with existing drugs), immunotherapy, and early antifungal chemotherapy driven by culture-independent biomarkers for BT-MCR and MCR in general [21]. The improvement of MCR diagnostics and the development of novel immune-enhancement strategies and agents against Mucorales remain the major unmet needs on the clinical research agenda for MCR [22].

## Figures and Tables

**Table 1:** Characteristics of patients with breakthrough mucormycosis (BT-MCR) on Mucorales-active versus other mold-active antifungals. Unless specified otherwise in the “characteristic” column, the number of patients and percentage (in parentheses) are given.

Characteristic		All Patients (*n* = 103)	Mucorales-Active Antifungals (*n* = 16)	Other Mold-Active Antifungals (*n* = 87)	*p*-Value
Age (years), median (range)		52 (18–76)	57 (25–75)	52 (18–76)	0.26
Gender, male		67 (65)	11 (69)	56 (64)	0.74
Race					0.45
	White	86 (84)	13 (81)	73 (84)	
	Black	11 (11)	3 (19)	8 (9)	
	Asian	6 (6)	0 (0)	6 (7)	
Ethnicity					0.12
	Hispanic	14 (14)	0 (0)	14 (16)	
	Non-Hispanic	89 (86)	16 (100)	73 (84)	
Underlying malignancy					>0.99
	Leukemia/MDS	92 (89)	15 (94)	77 (89)	
	Lymphoma/myeloma	11 (11)	1 (6)	10 (11)	
Status of primary disease					0.51
	Active	80 (78)	14 (88)	66 (76)	
	In remission	23 (22)	2 (12)	21 (24)	
Allogeneic HSCT recipients		50 (49)	5 (31)	45 (52)	0.13
GvHD		40/50 (80)	5/5 (100)	35/45 (78)	0.57
	Active	23/40 (58)	3/5 (60)	20/35 (57)	
	Chronic	17/40 (43)	2/5 (40)	15/35 (43)	
Neutropenia at diagnosis		65 (63)	12 (75)	53 (61)	0.28
Neutrophil recovery after neutropenia		41/65 (63)	6/12 (50)	35/53 (66)	0.33
Duration of neutropenia at diagnosis (days), median (IQR)		21 (12–52)	29 (20–49)	18 (11–54)	0.26
Corticosteroid use		36 (35)	5 (31)	31 (36)	0.74
History of diabetes mellitus		42 (41)	2 (13)	40 (46)	0.012
Type of infection					0.58
	Localized infection	19 (18)	2 (13)	17 (20)	
	Sinopulmonary infection	65 (63)	12 (75)	53 (61)	
	Disseminated infection	19 (18)	2 (13)	17 (20)	
Genus isolated					0.77
	*Rhizopus* spp.	61 (59)	10 (63)	51 (59)	
	*Mucor* spp.	18 (17)	3 (19)	15 (17)	
	*Rhizomucor* spp.	1 (12)	2 (13)	10 (11)	
	*Cunninghamella* spp.	8 (8)	0	8 (9)	
	*Absidia/Lichtheimia* spp.	4 (4)	1 (6)	3 (3)	
APACHE II score at diagnosis, median (IQR)		14 (12–17)	16 (13–18)	14 (12–17)	0.31
ICU admission at diagnosis		13 (13)	2 (13)	11 (13)	>0.99
ICU/Hospice at any time during treatment		79 (77)	15 (94)	64 (74)	0.11
Days from symptom onset to treatment initiation, median (IQR)		6 (3–11)	4 (2–10)	7 (3–12)	0.16
Initial treatment strategy					0.88
	AMB + POSA + CAS	31 (30)	5 (31)	26 (30)	
	AMB + POSA	26 (25)	6 (38)	20 (23)	
	AMB + CAS	21 (20)	2 (13)	19 (22)	
	AMB	1 (15)	2 (13)	13 (15)	
	AMB + ISA	8 (8)	1 (6)	7 (8)	
	POSA	2 (2)	0 (0)	2 (2)	
Surgical treatment of mucormycosis		47 (46)	3 (19)	44 (51)	0.019

Abbreviations: AMB = (lipid-formulation) amphotericin B, APACHE II = Acute Physiology And Chronic Health Evaluation II, CAS = caspofungin, GvHD = graft versus host disease, HSCT = hematopoetic stem cell transplant, ICU = intensive care unit, IQR = interquartile range, ISA = isavuconazole, MDS = myelodysplastic syndrome, POSA = posaconazole.

**Table 2:** Clinical characteristics of patients with breakthrough mucormycosis while receiving Mucorales-active antifungals.

Anti-Fungal	Year	Daily Dose	Form	Serum Level	Age/Sex	Cancer	Days ANC <500 ^a^	ANC <500 at Dx?	ANC Reco-Very ^b^	Antifungal Indication	Days AntiFungal ^c^	BT-MCR Location	Pathogen	Treatment Strategy	Day 42 Outcome ^d^
AMB	2004	5 mg/kg	IV	N/D	29/M	Burkitt’s	34	yes	no	Treatment ^e^	7	Localized	*Rhizopus* spp.	AMB	dead
ISA	2015	372 mg	tab	N/D	61/F	R/R AML	11	yes	yes	1º PPx (NP)	34	Pulmonary	*Mucor* spp.	AMB + PSOA	alive
ISA	2016	372 mg	tab	N/D	75/M	act AML	24	yes	no	1º PPx (NP)	14	Disseminated	*Rhizopus* spp.	AMB + POSA	dead
ISA	2016	372 mg	tab	N/D	47/M	act ALL	110	yes	yes	1º PPx	37	Sinusitis	*Rhizopus* spp.	AMB + POSA	dead
ISA	2016	372 mg	tab	N/D	61/M	R/R CLL	191	yes	no	Treatment ^f^	243	Pulmonary	*Rhizomucor* spp.	AMB + POSA	dead
ISA	2017	372 mg	tab	N/D	60/F	R/R ALL	47	yes	no	Treatment ^g^	151	Pulmonary	*Rhizopus* spp.	AMB + CAS + POSA	dead
ISA	2018	372 mg	tab	N/D	45/M	R/R CLL	0	no	-	1º PPx (GC)	60	Sinusitis	*Rhizopus* spp.	AMB + POSA	alive
ISA	2019	372 mg	tab	N/D	54/M	act AML	21	yes	no	1º PPx	106	Pulmonary	*Mucor* spp.	AMB + POSA	dead
ISA	2019	372 mg	tab	N/D	49/F	R/R AML	51	yes	yes	Treatment ^h^	16	Sinusitis	*Rhizopus* spp.	AMB + CAS + POSA	alive
ISA	2019	372 mg	tab	N/D	65/F	act AML	44	yes	no	Treatment ^i^	27	Sinusitis	*Rhizopus* spp.	AMB + CAS + POSA	dead
POSA	2000	800 mg	liquid	N/D	29/F	AML (CR)	0	no	-	Treatment ^j^	211	Disseminated	*Rhizopus* spp.	AMB	dead
POSA	2009	600 mg	liquid	N/D	52/M	CLL (CR)	0	no	-	1º PPx (GC)	61	Sinusitis	*Rhizopus* spp.	AMB + CAS	dead
POSA	2012	800 mg	liquid	685 ng/dL	70/M	R/R AML	20	yes	yes	1º PPx (NP) ^k^	54	Pulmonary	*Rhizomucor* spp.	AMB + CAS	dead
POSA	2016	300 mg	tab	1750 ng/dL	61/M	R/R AML	14	yes	yes	1º PPx (NP)	17	Cutaneous	*Rhizopus* spp.	AMB + CAS + POSA	alive
POSA	2017	300 mg	tab	2520 ng/dL	25/M	R/R ALL	20	yes	yes	1º PPx (NP)	437	Sinusitis	*Absidia* spp.	AMB + CAS + POSA	alive
POSA	2019	300 mg	tab	2180 ng/dL	68/M	act AML	0	no	-	1º PPx (NP)	63	Pulmonary	*Mucor* spp.	AMB + ISA	dead

^a^ Known days of ANC <500 until date of culture collection; ^b^ ANC >500 for at least 3 days during treatment; ^c^ days of medication until date of culture collection; ^d^ from treatment onset; ^e^ empiric treatment for pneumonia, which was found to be due to *Stenotrophomonas;* ^f^ treatment of aspergillosis; ^g^ empiric treatment of right pulmonary infiltrates during neutropenic fever (POSA not used due to elevated liver enzymes), then transitioned to prophylaxis; ^h^ empiric treatment after a positive galactomannan test from bronchoalveolar lavage—switched from POSA to ISA; ^i^ empiric treatment of neutropenic fever with concern for fungal pneumonia on chest CT—ISA used due to elevated liver enzymes; ^j^ treatment of *Alternaria* skin and soft tissue infection of lower extremities in prior month; ^k^ switched from voriconazole due to hallucinations. Abbreviations: act = active, ALL = acute lymphocytic leukemia, AMB = amphotericin B, AML = acute myeloid leukemia, ANC = absolute neutrophil count, BT-MCR = breakthrough mucormycosis, CAS = caspofungin, CLL = chronic lymphocytic leukemia, CR = complete remission, CT = computed tomography, Dx = diagnosis, F = female, GC = glucocorticoids, ISA = isavuconazole, IV = intravenous, M = male, mg = milligrams, N/D = not done, NP = neutropenia, POSA = posaconazole, 1º PPx = primary prophylaxis, R/R = relapsed/refractory, spp. = species, tab = tablets.

**Table 3:** Outcomes of patients admitted for BT-MCR while on Mucorales-active versus other mold-active antifungals by univariate analysis.

Characteristic	All Patients (*n* = 103)	Mucorales-Active Antifungals (*n* = 16)	Other Mold-Active Antifungals (*n* = 87)	*p*-Value
42-Day mortality from treatment initiation	45 (44)	11 (69)	34 (39)	0.028
84-Day mortality from treatment initiation	64 (62)	16 (100)	48 (55)	0.0007
42-Day mortality from symptom onset	32 (31)	10 (63)	22 (25)	0.006
84-Day mortality from symptom onset	61 (59)	16 (100)	45 (52)	0.0003
Days from treatment initiation to death, median (IQR)	44 (27–110)	27 (12–50)	49 (30–146)	0.007
Days from symptom onset to death, median (IQR)	55 (34–114)	35 (22–59)	61 (40–151)	0.007

**Table 4:** Cox regression model of independent predictors of 42-day mortality in patients with BT-MCR.

**(A) Mortality within 42 Days of Treatment Initiation**				
**Predictors**		**Hazard Ratio**	**95% Confidence Interval**	***p*-Value**
ICU at diagnosis		2.46	1.07 to 5.68	0.034
APACHE II score at diagnosis	Every 1-unit increase	1.21	1.12 to 1.30	<0.0001
Neutropenia status				<0.001
	No neutropenia at diagnosis	Reference		
	Neutropenia, recovered	0.83	0.37 to 1.84	
	Neutropenia, not recovered	3.25	1.53 to 6.90	
Antifungal prophylaxis				0.015
	Mucorales-active	2.40	1.19 to 4.86	
	Other mold-active	Reference		
**(B) Mortality within 42 Days of Symptom Onset**				
**Predictors**		**Hazard Ratio**	**95% Confidence Interval**	***p*-Value**
ICU at diagnosis		4.71	2.05 to 10.85	<0.001
APACHE II score at diagnosis	Every 1-unit increase	1.13	1.04 to 1.23	0.005
Neutropenia status				<0.0001
	No neutropenia at diagnosis	Reference		
	Neutropenia, recovered	0.67	0.23 to 2.00	
	Neutropenia, not recovered	9.63	3.57 to 25.99	
Antifungal prophylaxis				<0.001
	Mucorales-active	4.63	1.91 to 11.23	
	Other mold-active	Reference		
Treatment *				<0.0001
	Amphotericin B + caspofungin	8.15	3.09 to 21.48	
	Others	Reference		

* Treatment was a time-dependent variable. Abbreviations: APACHE II = Acute Physiology And Chronic Health Evaluation II, ICU = intensive care unit.

## Supplementary Materials

The following are available online at https://www.mdpi.com/2309-608X/7/3/217/s1, Table S1: Cox regression model of independent predictors of 84-day mortality in patients with breakthrough mucormycosis. Figure S1: Kaplan–Meier curves of progression to 84-day mortality in patients with hematologic malignancy and recipients of hematopoietic cell transplants with breakthrough mucormycosis on Mucorales-active versus other mold-active antifungals. Figure S2: Breakdown of survival outcomes of BT-MCR by individual (groups of) antifungal drugs. Figure S3: Kaplan–Meier curves of progression to all-cause 42-day and 84-day mortality of breakthrough mucormycosis, depending on the year of diagnosis.
