# Supplementary material for: Breakthrough Mucormycosis Developing on Mucorales-Active Antifungals Portrays a Poor Prognosis in Patients with Hematologic Cancer
Source: J Fungi (Basel). 2021 Mar 17;7(3):217. doi: 10.3390/jof7030217 (PMC8002622; doi:10.3390/jof7030217)
Supplement: Supplementary file 1 [file jof-07-00217-s001.pdf]

Journal of *Fungi*

MDPI

Article

Breakthrough Mucormycosis Developing on Mucorales-Active Antifungals Portrays a Poor Prognosis in Patients with Hematologic Cancer

Supplementary Materials

Table S1. Cox regression model of independent predictors of 84-day mortality in patients with breakthrough mucormycosis.

**(A) Mortality within 84 days of treatment initiation**			
**Predictors**	**Hazard ratio**	**95% Confidence Interval**	**p-value**
Cancer status			0.048
Active	2.50	1.01 to 6.21	
Remission	Reference		
ICU at diagnosis	2.98	1.37 to 6.47	0.006
APACHE II score at diagnosis (every 1-unit increase)	1.18	1.10 to 1.27	< .0001
Neutropenia status			< .0001
No neutropenia at diagnosis	Reference		
Neutropenia, recovered	0.71	0.35 to 1.44	
Neutropenia, not recovered	3.44	1.59 to 7.43	
Antifungal prophylaxis			< .001
Mucorales-active	3.29	1.76 to 6.15	
Other mold-active	Reference		
Treatment			< .001
Amphotericin B + caspofungin	3.22	1.68 to 6.17	
Others	Reference		

**(B) Mortality within 84 days of symptom onset**			
**Predictors**	**Hazard ratio**	**95% Confidence Interval**	**p-value**
Cancer status			0.039
Active	2.81	1.06 to 7.48	
Remission	Reference		
ICU at diagnosis	4.80	2.32 to 9.89	< .0001
APACHE II score at diagnosis (every 1-unit increase)	1.16	1.09 to 1.23	< .0001
Neutropenia status			< .0001
No neutropenia at diagnosis	Reference		
Neutropenia, recovered	0.82	0.40 to 1.70	
Neutropenia, not recovered	5.10	2.28 to 11.38	
Antifungal prophylaxis			< .0001
Mucorales-active	3.73	1.97 to 7.07	
Other mold-active	Reference		
Treatment*			< .001
Amphotericin B + caspofungin	3.57	1.84 to 6.91	
Others	Reference		

* Treatment was a time-dependent variable in the analysis. Abbreviations: ICU = intensive care unit.

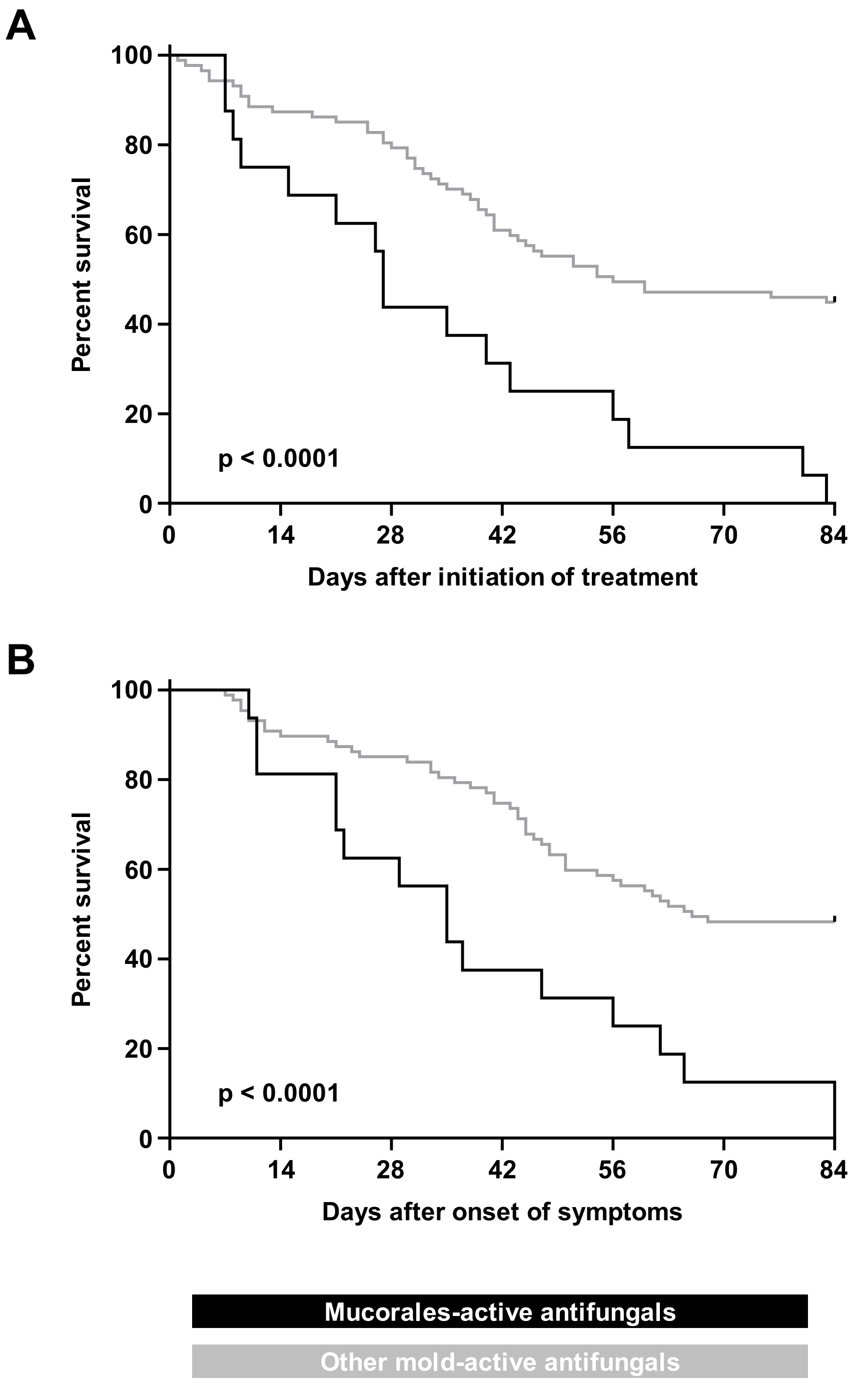

Figure S1. Kaplan Meier curves of progression to 84-day mortality in patients with hematologic malignancy and recipients of hematopoietic cell transplants with breakthrough mucormycosis on Mucorales-active versus other mold-active antifungals as measured from (**A**) initiation of treatment and (**B**) onset of symptoms.

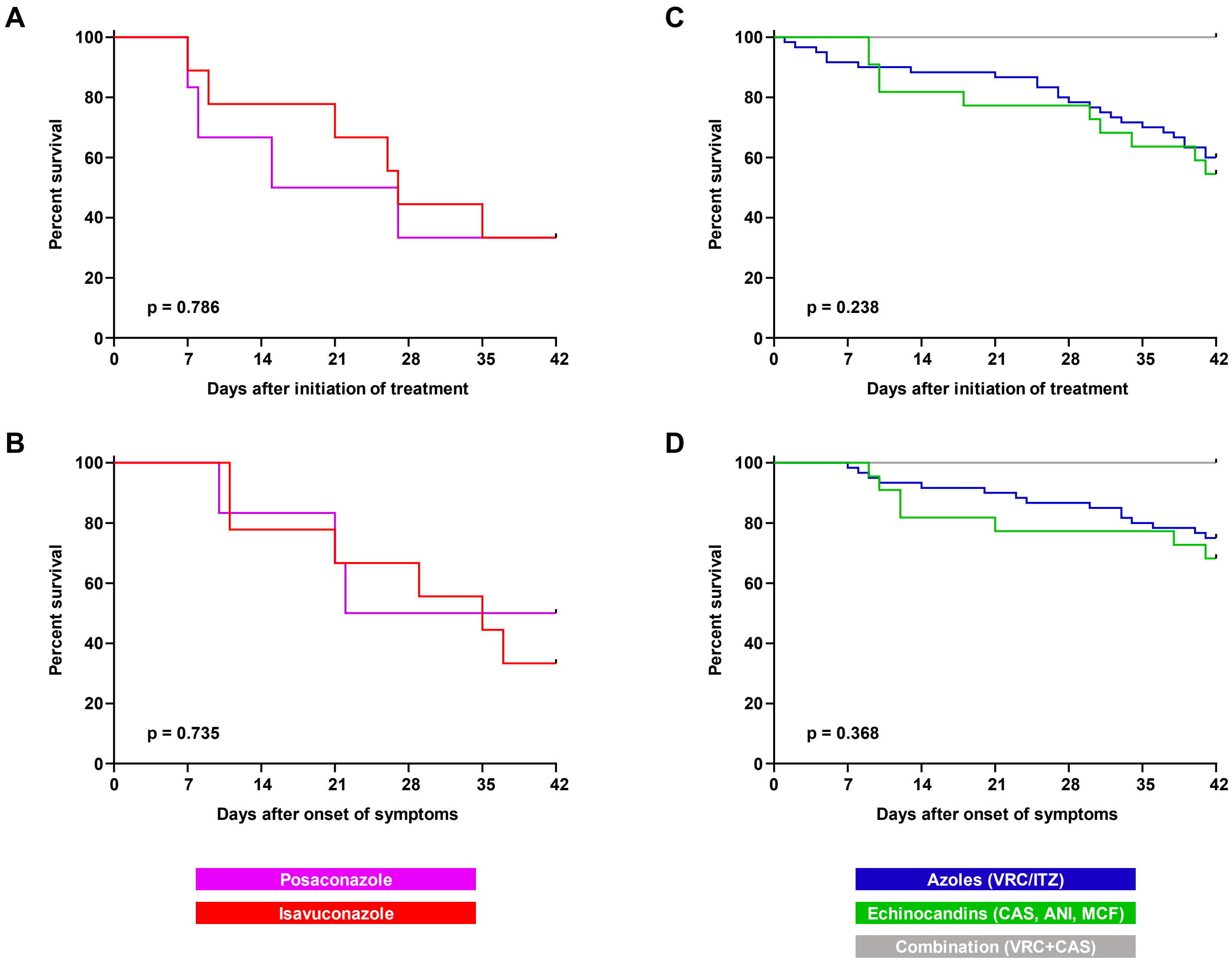

Figure S2. Breakdown of survival outcomes of BT-MCR by individual (groups of) antifungal drugs. Kaplan Meier curves show progression to 42-day mortality in patients with BT-MCR on Mucorales-active antifungals as measured from (**A**) initiation of treatment and (**B**) onset of symptoms; and other mold-active antifungals from (**C**) initiation of treatment and (**D**) onset of symptoms. The single patient with BT-MCR to amphotericin B has not been included in panels (**A**) and (**B**).

Abbreviations: BT-MCR = breakthrough mucormycosis, ANI = anidulafungin, CAS = caspofungin, ITZ = itraconazole, MCF = micafungin, VRC = voriconazole.

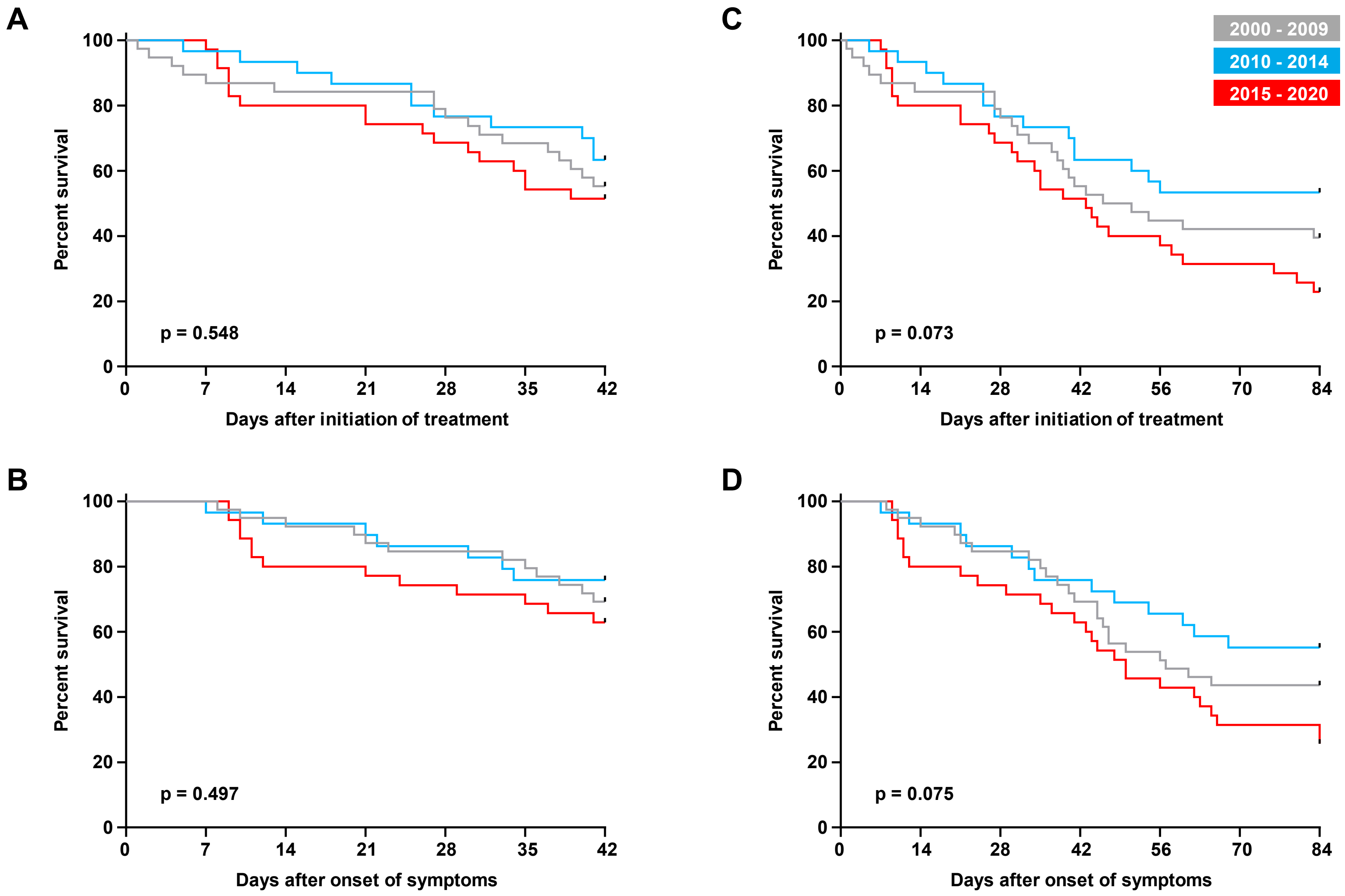

Figure S3. Kaplan Meier curves of progression to all-cause 42-day mortality of breakthrough mucormycosis from (**A**) initiation of treatment and (**B**) onset of symptoms, and 84-day mortality from (**c**) initiation of treatment and (**d**) onset of symptoms, depending on the year of diagnosis.
